# 纳米材料掺杂聚合物整体柱的构筑及在前处理领域的应用

**DOI:** 10.3724/SP.J.1123.2020.05030

**Published:** 2021-03-08

**Authors:** Ziling LI, Na LI, Tengwen ZHAO, Ziyang ZHANG, Manman WANG

**Affiliations:** 华北理工大学公共卫生学院, 河北 唐山 063210; School of Public Health, North China University of Science and Technology, Tangshan 063210, China; 华北理工大学公共卫生学院, 河北 唐山 063210; School of Public Health, North China University of Science and Technology, Tangshan 063210, China; 华北理工大学公共卫生学院, 河北 唐山 063210; School of Public Health, North China University of Science and Technology, Tangshan 063210, China; 华北理工大学公共卫生学院, 河北 唐山 063210; School of Public Health, North China University of Science and Technology, Tangshan 063210, China; 华北理工大学公共卫生学院, 河北 唐山 063210; School of Public Health, North China University of Science and Technology, Tangshan 063210, China

**Keywords:** 纳米材料, 聚合物整体柱, 样品前处理, 综述, nanomaterials, polymeric monolithic columns, sample pretreatment, review

## Abstract

聚合物整体柱是由单体、交联剂、引发剂和致孔剂在模具中通过原位聚合而成的棒状整体。与传统的填充式固相萃取柱相比,聚合物整体柱吸附剂凭借制备简单、柱压低、传质快及pH使用范围宽泛等优点已广泛应用于食品分析、生物医药和环境卫生等领域的前处理中。然而,通常由于聚合方式难以控制,聚合物整体柱在制备过程中容易产生颗粒堆积、孔道不均匀从而导致孔隙率低和比表面积有限等问题。近年来,将纳米材料掺杂至聚合物整体柱以获得有序结构分布、良好吸附效率以及优越选择性能的新型吸附剂成为研究热点。纳米材料种类繁多,尺寸小,利用其表面丰富的活性基团、作用位点和超大的比表面积等优势,通过简单掺杂、共聚合和原位修饰等方法合成纳米掺杂聚合物整体柱,不仅能够改善其微观结构、柱床机械强度和稳定性,同时可以显著提高掺杂聚合物整体柱吸附剂的萃取性能和选择性。该文综述了碳材料、金属和金属氧化物、金属有机骨架、共价有机骨架和无机纳米粒子等不同纳米材料掺杂的聚合物整体柱、常用的构筑方法以及该类吸附剂在固相萃取、固相微萃取、搅拌棒吸附萃取和在线固相萃取等样品前处理领域的应用,并展望这一研究的发展趋势和应用前景,以期为前处理领域的研究提供参考。

## Abstract (en)

Polymeric monolithic columns are fabricated by in situ polymerization of the corresponding monomer, crosslinkers, porogenic solvents and radical initiators within a mold. Compared with the conventional packed solid phase extraction adsorbents, polymeric monolithic columns with a continuous porous structure process distinctive advantages of rapid mass transfer and excellent permeability, which facilitates the extraction of trace amounts of the target from the matrix even at high flow velocities. Besides, these materials can be easily fabricated in situ within various cartridges, avoiding a further packing step associated with packed particulate adsorbents. Additionally, the abundant monomer availability, flexible porous structure, and wide applicable pH range make monoliths versatile for use in separation science. Thus, polymeric monolithic columns have been increasingly applied as efficient and promising extraction media for sample pretreatment food, pharmaceutical, biological and environmental analyses. However, these materials usually have the difficulty in morphology control and their interconnected porous micro-globular structure, which may result in low porosity, limited specific surface area and poor efficiency. In addition, polymeric monoliths suffer from the swelling in organic solvents, thus decreasing the service life and precision while increasing the cost consumption. Recently, the development of nanomaterial-incorporated polymeric monoliths with an improved ordered structure, enhanced adsorption efficiency and outstanding selectivity has attracted considerable attention. Nanoparticles are considered as particulates within the size range of 1-100 nm in at least one dimension, which endows them with unique optical, electrical and magnetic properties. These materials have a large surface area, excellent thermal and chemical stabilities, remarkable versatility, as well as a wide variety of active functional groups on their surface. With the aim of exploiting these advantages, researchers have shown great interest in applying nanomaterial-incorporated polymeric monoliths to separation science. Accordingly, significant progress has been achieved in this field. Nanomaterials can be entrapped via the direct synthesis of a polymerization solution that contains well dispersed nanomaterials in porogens. In addition, nanoparticles can be incorporated into the monolithic matrix by copolymerization and post-polymerization modification via specific interactions. Therefore, nanomaterial-incorporated polymeric monoliths combined the different shapes, chemical properties, and physical properties of the polymers with those of the nanoparticles. The presence of nanoparticles can improve the structural rigidity as well as the thermal and chemical stabilities of monolithic adsorbents. Besides, nanoparticles are capable of increasing the specific surface area and providing multiple active sites, which leads to enhanced extraction performance and selectivity of polymeric monolithic materials. In recent years, diverse types of nanomaterials, such as carbonaceous nanoparticles, metallic materials and metal oxides, metal-organic frameworks, covalent organic frameworks and inorganic nanoparticles have been extensively explored as hybrid adsorbents in the modes of solid phase extraction, solid phase microextraction, stir bar sorption extraction and on-line solid phase extraction. This review specifically summarizes the fabrication methods for nanomaterial incorporated polymeric monoliths and their application to the field of sample pretreatment. The existing challenges and future possible perspectives in the field are also discussed.

聚合物整体柱由Hjertén等^[1]^在1989年提出,是由单体、交联剂、引发剂和致孔剂在模具中通过原位聚合而成的棒状整体。与传统的填充柱不同,聚合物整体柱制备简单,形式灵活,特有的“连续”结构赋予其通透性好、柱压低和传质快的优势,另外,有机聚合物整体柱生物兼容性良好及pH使用范围宽泛。基于以上诸多优势,聚合物整体柱在分离分析方面备受瞩目,特别是作为一种吸附剂材料,已广泛应用于食品分析、生物医学和环境卫生等领域的前处理中^[2,3,4,5]^。

然而,聚合物整体柱的聚合方式难以控制,在制备过程中容易产生颗粒堆积结构,从而导致孔隙率低和比表面积有限等问题。此外,聚合物整体柱在有机溶剂中易发生溶胀,会影响其使用寿命和整个方法的精密度。近年来,将纳米材料掺杂至聚合物整体柱,发展具有有序结构分布、良好吸附效率以及优越选择性能的新型吸附剂成为研究热点^[6,7]^。

本文综述了纳米材料掺杂聚合物整体柱的构筑及在样品前处理领域的应用。

## 1 纳米材料掺杂聚合物整体柱

纳米材料是指在三维空间中至少有一维处于纳米级(1~100 nm)或由纳米级为基本单元构成的材料,从而在光学、电学、磁学和化学等方面具有奇异的特性。纳米材料种类繁多,尺寸小,具有更大的比表面积和更多样的表面性能^[8,9,10]^。目前,碳纳米管(carbon nanotube, CNT)、石墨烯(graphene, G)、金/银纳米粒子、金属有机骨架(metal organic frameworks, MOFs)和共价有机骨架(covalent organic frameworks, COFs)等已成功掺杂到聚合物整体柱中(见图1),一方面这些材料表面丰富的活性基团、作用位点及超大的比表面积可以提高聚合物整体柱的萃取性能或选择性,另一方面,能够改善原有的颗粒堆积和孔道不均匀等问题,提高柱床的机械强度和稳定性^[11,12,13]^。

**图1:**
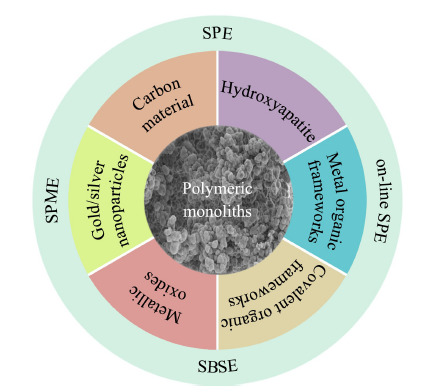
纳米材料掺杂聚合物整体柱的示意图

### 1.1 碳材料

碳的多种同素异形体使其表现出结构的多样性,例如,零维的富勒烯、一维的碳纳米管和二维的石墨烯以及多孔碳材料活性炭。碳材料具有良好的化学稳定性、比表面积大和吸附容量高等优点,特别是本身表面超大的*π*电子共轭体系能够与一些化合物和金属离子产生*π-π*共轭和疏水作用,这些优异特性使得碳材料成为吸附和分离领域活跃的家族^[14,15]^。

1.1.1 碳纳米管

CNT是由石墨烯片层卷曲而成的一维碳纳米材料,根据石墨烯层数可分为单壁碳纳米管(single-walled carbon nanotube, SWNT)和多壁碳纳米管(multi-walled carbon nanotube, MWNT)。SWNT是由单层碳原子组成,直径为0.4~3.0 nm;而MWNT是由多个同心管组成,直径可达100 nm。CNT表面含有丰富的*π*电子,具有疏水性强、化学稳定性和热稳定性良好等优点,比表面积可达500 $m^{2}/g^{}$ ^[16,17]^。

Fresco-Cala等^[18]^以甲基丙烯酸缩水甘油酯(GMA)为单体,乙二醇二甲基丙烯酸酯(EDMA)为交联剂,首次通过光聚合法在移液器枪尖中制备聚(MWNT-GMA-co-EDMA)固相微萃取整体柱,发展了简单、高效且成本低廉的生物样品中抗抑郁药的前处理方法。MWNT的*π-π*作用,提高了整体柱对抗抑郁药的萃取效率。Yu等^[19]^制备了以MWNT和苯乙烯(styrene, St)为共聚单体、EDMA为交联剂的整体柱,用于固相萃取前处理植物药材中的熊果酸。掺杂MWNT后,聚合物整体柱的比表面积从12.75 m^2^/g提升至43.22 m^2^/g,对熊果酸的吸附容量由20 mg/g显著提升至50 mg/g,但是MWNT的弱极性会引起整体柱制备过程中聚合溶液分散困难的问题,有效解决方法是将MWNT羧基化处理后再掺杂。Makkliang等^[20]^以甲基丙烯酸(methacrylic acid, MAA)为单体,EDMA为交联剂,将羧基化的MWNT掺杂到聚(MAA-co-EDMA)整体柱,作为SPME吸附剂前处理化妆品和个人护理品中4种对羟基苯甲酸,建立了一种成本低廉的萃取装置,实现了同时、快速萃取6种实际样品。羧基化MWNT改善了制备过程中的分散性,且表面*π-π*共轭和氢键作用将整体柱对4种对羟基苯甲酸的萃取效率提高至100%,明显高于空白柱的53.5%~91.8%。方法的检出限为0.63~0.80 ng/mL,回收率为83.4%~102.9%,该柱可重复使用至少15次,相对标准偏差≤5.2%。

1.1.2 石墨烯

G是由碳原子紧密堆积成单层二维蜂窝状晶格结构的一种碳质新材料,紧密堆积方式和*sp*^2^杂化成键连接的特点赋予其独特性质,与碳纳米管相比,G具有超大的比表面积,理论计算值达2630 m^2^/g,因而自2014年首次获得以来,在生物传感、药物传输和分离科学等领域掀起了研究热潮^[21,22]^。

Tong等^[23]^将G掺杂至甲基丙烯酸丁酯(butyl methacrylate, BMA)为单体、EDMA为交联剂的整体柱,G的疏水、*π-π*共轭和氢键相互作用增强了掺杂整体柱对糖皮质激素的富集能力,结合高效液相色谱-串联质谱法测定了化妆品中糖皮质激素,检出限为0.13~1.93 ng/mL,回收率为83.7%~103.8%。Pei等^[24]^以4-乙烯基吡啶(4-vinylpyridine, VP)为功能单体、EDMA为交联剂,采用热引发聚合法制备聚(G-VP-co-EDMA)毛细管整体柱前处理环境水和大米中的苯氧乙酸除草剂。与空白柱相比,G加入后将比表面积由41.5 m^2^/g增加至136.2 m^2^/g,通过*π-π*、疏水、离子交换和氢键作用萃取目标物,对苯氧乙酸的富集能力提高了2.2~2.9倍,方法简便、灵敏且环保,为复杂样品基质中苯氧乙酸除草剂的检测提供了新思路。

氧化石墨烯(graphene oxide, GO)是石墨烯重要的衍生物,由于它在单层碳原子构成的二维空间无限延伸的表面含有大量的含氧基团,如羟基、羧基、环氧基和羰基等,因此可以在水和其他多种有机溶剂中较好地分散,亲水性佳,生物兼容性好^[25,26]^。

Tong等^[27]^制备了聚(GO-GMA-co-EDMA)整体柱,作为SPE吸附剂,结合液相色谱-串联质谱法测定尿液中肌氨酸。方法的检出限为1.0 ng/mL,回收率为78.5%~96.2%,聚(GO-GMA-co-EDMA)整体柱可对肌氨酸富集32倍。Jing等^[28]^制备聚(GO-MAA-co-EDMA)整体柱,结合高效液相色谱法,在线SPME前处理环境水中氨基甲酸酯杀虫剂,GO的引入提高了整体柱对目标物的富集能力,检出限为0.3~0.7 μg/L,回收率为78.9%~103.4%。本课题组^[29]^制备了聚(GO-EDMA)整体柱作为在线SPE净化柱,与高效液相色谱-串联质谱联用,实现了牛奶和鸡肉中16种磺酰胺类药物的快速和自动化测定。GO掺杂后,整体柱的比表面积由65 m^2^/g增加至226 m^2^/g,整个方法分析时间为23 min,在牛奶和鸡肉中的检出限分别为0.3 μg/kg和0.6 μg/kg,回收率分别为70.3%~98.5%和79.0%~108.0%。

1.1.3 活性炭

活性炭(active carbon, AC)是多孔结构的碳材料在厌氧或无氧环境下经高温热解活化产生的材料,具有机械强度高、化学性质稳定、耐酸碱和耐热、丰富的孔隙结构、巨大的比表面积(500~1700 m^2^/g)、表面含有羧基、羟基和羰基等活性基团的特性。另外,AC来源广泛,制备简单,成本低廉,可再生使用,因此已在复杂样品基质前处理领域中展现出良好的应用潜力^[30,31]^。

Lirio等^[32]^通过直接掺杂法在毛细管中制备聚(AC-BMA-co-EDMA)和聚(AC-St-co-二乙烯基苯(divinylbenzene, DVB))整体柱,作为SPME吸附剂,结合超高液相色谱-紫外检测,分析水中5种邻苯二甲酸酯。AC的掺杂改变了聚合物整体柱表面极性,增强了柱的亲水性,聚(AC-BMA-co-EDMA)对邻苯二甲酸酯的萃取效率由空白柱的15.9%~53.1%增长至54.2%~79.7%,聚(AC-St-co-DVB)提升至76.2%~99.3%,且可重复使用30次。Kuo等^[33]^通过微波辅助法制备聚(AC-BMA-co-EDMA)毛细管整体柱,用于果酒和蔓越莓果汁中酚酸的前处理。由于AC和酚酸之间产生的氢键和*π-π*共轭作用,掺杂AC的聚合物整体柱能够同时萃取9种酚酸,且萃取效率由2.0%~49.9%提升至32.5%~78.8%,展现了掺杂聚合物整体柱富集净化酚酸类化合物的应用潜力。

综上,碳材料掺杂聚合物整体柱研究报道很多,主要是依靠碳材料本身*π-π*共轭作用改善聚合物整体柱的性能。制备过程中,碳纳米管和石墨烯由于自身的弱极性,在制备掺杂聚合物整体柱时,在聚合溶液中的分散性是首要面对的问题,在选择聚合单体和致孔剂、溶剂时相对苛刻。将它们表面进行衍生后,聚合条件变得宽松,获得的聚合物整体柱也更多样化。

### 1.2 金属和金属氧化物

1.2.1 金/银纳米粒子

金/银纳米粒子是由金/银原子组成的纳米颗粒,其粒径通常为1~100 nm,具有较高的比表面积和良好的生物相容性。金/银纳米粒子与巯基和氨基等基团的特异性结合,基于这一特点,一方面可用于构筑金/银纳米粒子掺杂的聚合物整体柱,还可以在应用时选择性富集含有特定基团的分析物^[34]^。

Mompó-Roselló等^[35]^制备聚(Au-GMA-co-EDMA)整体柱,结合高效液相色谱-荧光检测法,分析尿液和唾液中的谷胱甘肽(见图2)。利用金纳米粒子表面的氨基和硫醇基对含有巯基非芳香族化合物具有强亲和力,聚(Au-GMA-co-EDMA)整体柱对谷胱甘肽的吸附容量为2.93 mg/g,富集倍数为5.8,方法的检出限达到1.53 ng/mL。聚合物整体柱重复使用15次后,对谷胱甘肽的回收率仍大于90%。Jiang等^[36]^制备了银纳米粒子掺杂的聚合物整体柱,该柱以透明质酸盐功能化的脲醛为功能单体,用于SPME前处理炸薯条中的单不饱和脂肪酸甲酯。掺杂后整体柱的比表面积由6.50 m^2^/g提升至26.78 m^2^/g。

**图2:**
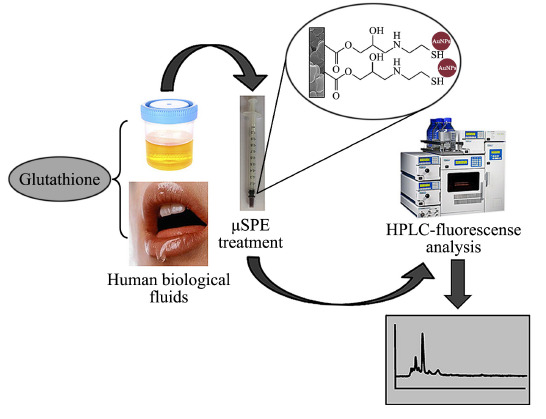
聚(Au-GMA-co-EDMA)整体柱SPE前处理尿液和唾液中谷胱甘肽的流程图

1.2.2 金属氧化物

金属氧化物是指由金属元素与氧元素组成的二元化合物,例如In_2_O_3_、ZnO、Fe_3_O_4_和Al_2_O_3_等。金属氧化物因独特的表面活性位点和特殊的物理化学性质,将其掺杂至整体柱内,一方面,表面的羟基能够与特定官能团相互作用,可以提供较多的活性位点吸附目标物,另一方面,金属氧化物良好的生物相容性和化学稳定性,使其成为掺杂聚合物整体柱的理想材料^[37]^。

Qi等^[38]^将In_2_O_3_与GMA和MAA单体聚合,制备了聚(In_2_O_3_-GMA-MAA-co-EDMA)整体柱,作为SPME吸附剂用于食品中12种人工色素的前处理。由于In_2_O_3_的引入,聚合物整体柱的机械性能和热稳定性显著提高,热分解的最高温度(440.5 ℃)明显高于空白柱(273.6 ℃)。另外,In_2_O_3_大的比表面积和表面丰富的羟基能够与罗丹明B、喹啉黄和日落黄等12种人工色素之间产生氢键作用,显著提高了掺杂柱的富集能力。Liu等^[39]^将ZnO纳米粒子引入聚(MAA-co-EDMA)整体柱,用于萃取环境水中4种氟喹诺酮类抗生素。ZnO能够与目标物产生络合和静电作用,成功地将萃取效率提升8.5倍,该方法简便、实用,适用于多种实际样品中氟喹诺酮的前处理。Krenkova等^[40]^利用静电作用将Fe_3_O_4_掺杂至聚(GMA-EDMA)表面,用于选择性富集磷酸肽,将*α*-酪蛋白酶解液中磷酸肽由未富集的5种增加至19种。Li等^[41]^将*γ*-Al_2_O_3_引入聚(*N*-异丙基丙烯酰胺-co-GMA-co-EDMA)毛细管整体柱中,用于萃取红酒中的苏丹染料。掺杂*γ*-Al_2_O_3_后,聚合物整体柱的富集能力显著提升15.9~18.7倍。

### 1.3 金属有机骨架

MOFs是由金属离子或离子簇与有机配体以高度有序的方式连接而成的一种晶体材料。MOFs的金属离子和有机配体种类繁多,且配位方式多样,决定了MOFs组成和结构的多样化,同时MOFs具有比表面积大、孔径分布均匀、表面性质可调等特点,已在气体存储、药物传输和吸附分离领域展现出良好的应用潜力^[42,43,44]^。

Lyu等^[45]^通过原位聚合法将MIL-53(Al)掺杂到聚(BMA-co-EDMA)整体柱中,结合高效液相色谱法,测定水和尿液中非甾体类抗炎药。掺杂MIL-53后,聚合物整体柱的比表面积从10.8 m^2^/g提升至107.6 m^2^/g,重复使用120次以上萃取性能无明显变化。Pang等^[46]^采用热引发聚合法制备MOF-199和*N*-羟甲基丙烯酰胺为共聚单体的聚合物整体柱,结合高效液相色谱,建立草药中熊果酸的分析方法。掺杂MOF-199后,聚合物整体柱的比表面积由空白柱的8.73 m^2^/g提升至18.29 m^2^/g,对熊果酸的吸附容量由15.14 mg/g增加至37.29 mg/g。Lin等^[47]^通过微波辅助法制备了聚(MIL-101-BMA-co-EDMA)毛细管整体柱,作为SPME吸附剂,实现了河水中6种青霉素的有效富集。MIL-101(Cr)加入后,通过路易斯酸碱和*π-π*共轭相互作用吸附青霉素,方法检出限能够达到1.2~4.5 ng/mL,且该柱可重复使用45次以上。

### 1.4 共价有机骨架

COFs是由C、H、O、N和B等轻质元素以共价键连接的二维或三维晶体多孔材料。COFs具有比表面积大、孔径可调、种类和性质多样、低密度和表面可功能化修饰等特点。此外,以B-O、C=N和C-N为主的共价键使COFs具有良好的化学和热稳定性。目前COFs广泛用于催化、光学器件和样品前处理等领域^[48,49]^。

Wang等^[50]^将COF表面修饰巯基,与GMA表面环氧基共价键结合制备了COF掺杂的聚(GMA-EDMA)毛细管整体柱,结合高效液相色谱-紫外检测,分析血液中的二苯甲酮(见图3)。COF掺杂的整体柱与二苯甲酮之间存在*π-π*和氢键作用,可有效富集二苯甲酮17.5~40.3倍。该整体柱重复使用50次,萃取效率无明显变化,且柱床无明显塌陷。Li等^[51]^合成了对非甾体抗炎药有高效富集能力的聚(COF-苯乙烯-二乙烯基苯-GMA)整体柱,在注射器中预浓缩水中7种非甾体抗炎药。掺杂COF的整体柱对目标物的吸附性能明显优于空白柱和商品化C_18_吸附剂,方法在1 min内即可达到吸附平衡,为环境水中非甾体抗炎药或其他极性污染物的快速提取提供了新思路。

**图3:**
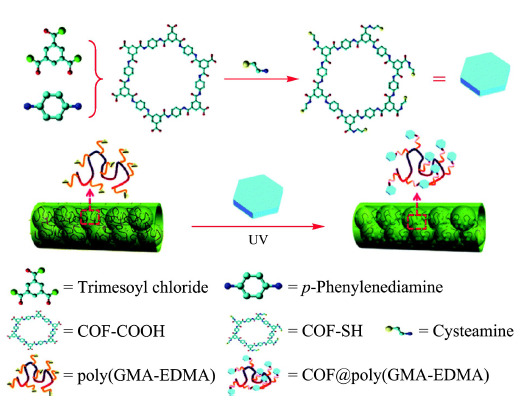
聚(COF-GMA-EDMA)整体柱的制备示意图

### 1.5 无机纳米材料

二氧化硅(SiO_2_)是以硅氧四面体为基本结构形成的立体网状结构,有大比表面积、较高的机械强度、良好的生物相容性和稳定的物理化学性质等优点;表面含有丰富的硅羟基,可以通过化学接枝对其表面改性,通过改性新增的基团增加与目标物的相互作用。基于此,SiO_2_在分离、样品前处理、催化和生物医药等领域具有良好的应用潜力^[52]^。

Xu等^[53]^以丙烯酰胺和*N*,*N*-亚甲基-双丙烯酰胺为单体,通过共聚法制备SiO_2_纳米粒子掺杂的聚合物整体柱分离和吸附蛋白质。SiO_2_纳米粒子的引入使得聚合物整体柱的孔隙率、渗透性和机械强度显著改善。

羟基磷灰石(hydroxyapatite, HAP)是一类钙磷类化合物,分子式为Ca_10_(PO_4_)_6_(OH)_2_,结构为六角柱体。作为人体骨骼组织的主要无机成分,HAP具有良好的生物相容性和生物活性,利用其表面的Ca^2+^和OH^-^可被其他金属离子置换的性质,目前已在去除环境水中无机金属离子、有机染料和吸附分离蛋白质方面广泛应用^[54,55]^。

Krenkova等^[56]^通过原位聚合法将HAP掺杂至聚(2-羟乙基甲基丙烯酸酯-co-EDMA)中,用于蛋白质的分离和磷酸肽的选择性富集。由于HAP表面Ca^2+^与磷酸肽具有强亲和力,HAP的掺杂增强了整体柱对磷酸肽的选择性,能够有效富集两种磷酸肽。Wang等^[57]^以脲醛为单体、聚乙二醇6000为致孔剂,采用一步缩聚反应制备HAP掺杂的聚合物整体柱,结合高效液相色谱-二极管阵列检测,建立了分析草鱼中三磷酸腺苷及其磷酸化代谢物的新方法,利用HAP和脲醛的亲水和离子交换作用,对三磷酸腺苷及其磷酸化代谢物实现良好的净化和富集效果,杂质峰的响应值降低6倍。检出限达到0.01~0.04 μg/g,回收率为78.3%~92.5%。

## 2 纳米材料掺杂聚合物整体柱的构筑方法

### 2.1 直接掺杂法

起初,纳米材料主要是通过简单掺杂法引入到聚合物整体柱中,即将纳米材料、单体、交联剂和引发剂在致孔剂中超声混合均匀,注入模具内,通过热或光引发聚合构筑聚合物整体柱。本课题组^[29]^在不锈钢柱内通过热引发自由基聚合直接制备了聚(GO-EDMA)整体柱,作为在线SPE吸附剂,开发了简单、快速的鸡肉和牛奶中16种磺酰胺类药物的分析方法(见图4)。与空白柱相比(比表面积为65 m^2^/g), GO掺杂的整体柱结构更均匀,具有更大的比表面积(226 m^2^/g)。同时,提高了该柱的稳定性,重复使用450次后,对磺酰胺类药物的萃取效率无明显改变,相对标准偏差≤11.8%。Kuo等^[33]^将AC与BMA、EDMA和致孔剂混合,超声处理后,填充于毛细管内热引发,用于固相微萃取食品中的酚酸。AC的引入使整体柱的比表面积由7 m^2^/g显著增加至352 m^2^/g,该柱至少重复使用9次。Zhang等^[58]^以光引发原位聚合制备了聚(*γ*-Al_2_O_3_-MAA-co-EDMA)整体柱,用于固相微萃取氯唑沙宗片中的2-氨基-4-氯苯酚。掺杂*γ*-Al_2_O_3_的整体柱的比表面积增加,对4-氯苯酚的富集倍数由7.3提升至17.0。

**图4:**
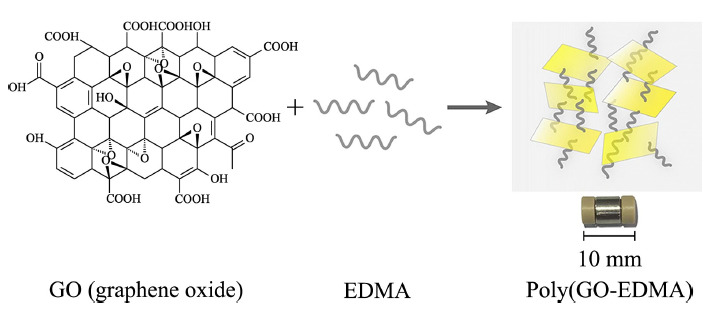
聚(GO-EDMA)整体柱的制备示意图

以纳米材料直接掺杂方式构筑聚合物整体柱,制备简单直接,无须复杂的化学修饰过程,但纳米材料与单体之间没有化学键合,通常易导致纳米材料分布不均和纳米材料流失等问题,同时,纳米材料也会包埋在柱床中,从而限制其有效使用,掺杂过量会导致聚集甚至沉淀,无法达到良好的萃取效果。另外,直接掺杂法对聚合溶液的种类和极性有一定条件要求,对于粒径大、密度大或表面极性弱等在聚合溶液中分散困难的纳米材料存在局限性,难以通过这一方法实现掺杂。

### 2.2 共聚法

共聚法是将纳米材料与单体通过化学键合或在纳米材料上修饰活性功能基团,再与交联剂聚合的方法。Luo等^[59]^在MIL-53(Al)表面修饰氨基后,与MAA表面的羧基结合形成酰胺键,热引发原位聚合法制备MIL-53(Al)掺杂的聚(St-DVB-MAA)毛细管整体柱,用于在线固相微萃取尿液中的雌激素。掺杂后的整体柱对雌激素的富集倍数高达180~304,且可重复使用100次。Giesbers等^[60]^在MIL-101表面修饰氨基与GMA表面环氧基共价键结合,通过原位聚合法制备聚(MIL-101-GMA-co-EDMA)整体柱,对尿液中3种非甾体类抗炎药进行前处理。掺杂MIL-101后的整体柱重复使用50次后,对非甾体类抗炎药的回收率仍大于75%。Liu等^[61]^将1,3,5-三羟基均苯三醛与甲基丙烯酸酐(methacrylic anhydride, MA)通过酯化反应结合,构建含有MA的COF材料为单体、EDMA为交联剂的聚合物整体柱,COF掺杂的整体柱结构均匀,通透性好,在分离多环芳烃、酚类和胺类化合物等有机小分子方面分离度和精密度良好。

使用共聚法制备的纳米材料掺杂整体柱中纳米材料可以和聚合物柱床通过共价键连接,结构更稳定,但是在合成中首先需要对单体进行功能化,同时对聚合条件需要优化,相比直接掺杂法略显复杂。

### 2.3 后修饰聚合法

后修饰方法是先合成不添加纳米材料的聚合物整体柱,再经一定方法将纳米材料修饰到整体柱表面。该方法主要依赖聚合物整体柱单体本身的官能团进行反应,由于GMA表面的活性环氧基团可以与巯基、氨基等基团反应,而成为最常用的聚合单体。Mompó-Roselló等^[35]^使用二苯甲酮和EDMA对聚丙烯注射器内表面进行改性,实现与整体柱柱床共价连接,通过光引发自由基聚合法制备聚(GMA-co-EDMA)整体柱,表面分别修饰氨基、半胱胺和胱胺后,将金纳米粒子与修饰的官能团结合掺杂,制备聚(Au-GMA-co-EDMA)整体柱对尿液和唾液中谷胱甘肽进行前处理(见图5)。整体柱重复使用15次后,对谷胱甘肽的回收率仍大于90%。张爱珠等^[62]^在毛细管中制备聚(GMA-co-EDMA)整体柱,表面修饰巯基后,利用金纳米粒子与巯基结合将其固定在聚合物整体柱表面,合成金纳米粒子掺杂的聚合物整体柱作为SPME吸附剂,萃取血浆中谷胱甘肽。由于金纳米粒子的引入,整体柱可对血浆中谷胱甘肽有效富集30倍。Jiang等^[36]^首先制备了透明质酸钠高性能化脲醛整体柱,使用脲甲醛进行修饰,银纳米粒子与脲甲醛相互作用,开发了银纳米粒子掺杂的聚合物整体柱,分析炸薯条中的单不饱和脂肪酸甲酯。掺杂后整体柱的比表面积由6.50 m^2^/g提升至26.78 m^2^/g,重复使用100次后,目标物的峰面积无明显改变。

**图5:**
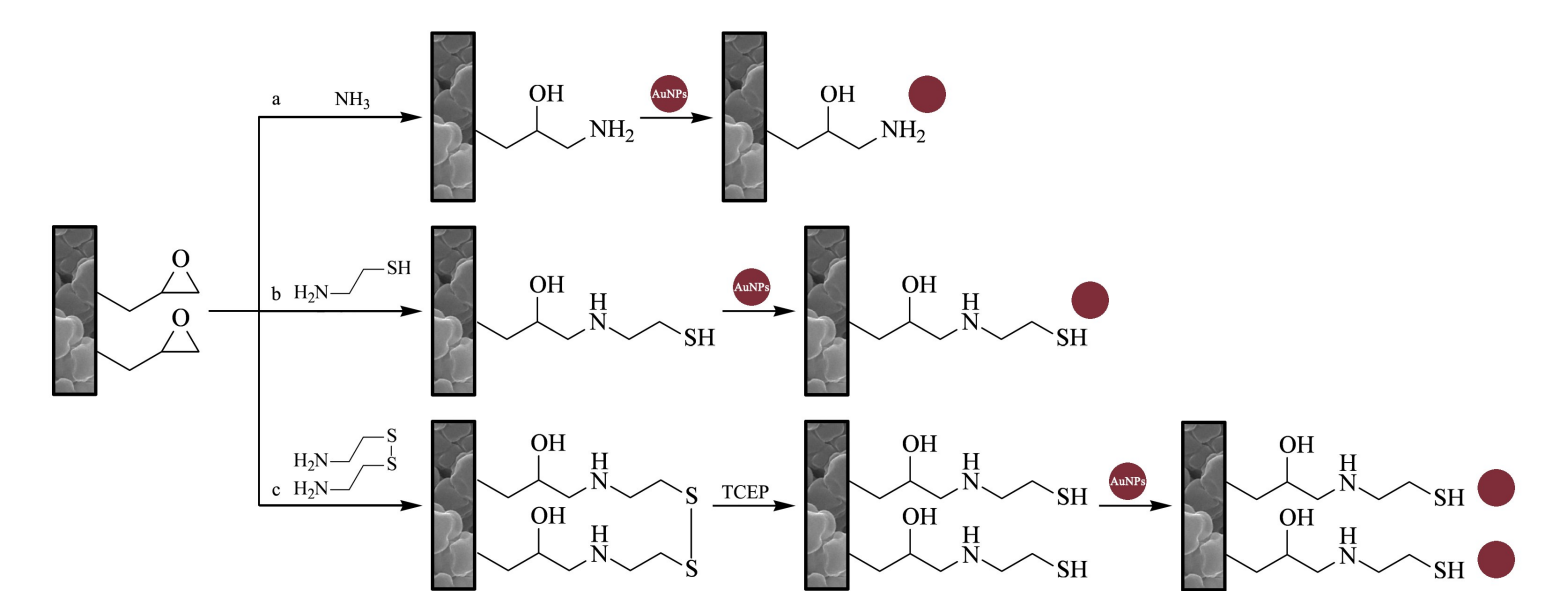
聚(GMA-co-EDMA)整体柱表面分别修饰(a)氨基、(b)半胱胺和(c)胱胺后与金纳米粒子结合的示意图^[35]^

通过后修饰法制备聚合物整体柱,将纳米材料化学键合到基体表面,获得的整体柱性质稳定,表面均匀,纳米材料的覆盖率更佳,但是需要控制聚合物整体柱载体的孔结构和纳米材料的覆盖率。

## 3 纳米材料掺杂聚合物整体柱在前处理领域的应用

样品前处理是指从复杂样品基质中净化和富集目标物,使原始样品中的待测组分转化为适用于仪器分析测定的形式,是分析过程中重要且必要的环节。样品前处理过程直接影响分析方法的精密度、选择性、可靠性以及分析成本,成为制约检测技术的瓶颈^[63,64,65]^。

作为样品前处理技术的核心,吸附剂决定了前处理的选择性和效率。纳米材料掺杂聚合物整体柱同时具备纳米材料比表面积大和吸附性能好的优良性能以及聚合物整体柱结构连续多孔、通透性好、柱压低和传质快的优点,通过SPE、SPME、搅拌棒吸附萃取(SBSE)和在线SPE等模式成功用于生物医药、环境卫生和食品安全领域样品前处理。

### 3.1 固相萃取

SPE技术是利用样品在两相(吸附剂和溶剂)之间分配系数的差异有效地将目标化合物与复杂基体分离,实现目标物净化与富集的一种前处理技术^[66,67,68]^。SPE具有有机溶剂消耗少和萃取效率高等优势,近年来,基于纳米材料掺杂的聚合物整体柱作为固相萃取吸附剂在样品前处理领域已成为研究热点。

Al-Rifai等^[69]^以甲基丙烯酸苄酯(benzyl methacrylate, BzMA)为单体、EDMA为交联剂,制备聚(CNT-BzMA-co-EDMA)整体柱,作为SPE吸附剂,结合高效液相色谱,测定水中多环芳烃。CNT的掺杂使整体柱对多环芳烃的萃取效率提升了48.6%~78.4%,富集倍数高达100倍,方法检出限为0.02~0.22 μg/L。本课题组^[70]^将GO与EDMA直接聚合,在注射器柱管中热引发制备了聚(GO-EDMA)整体柱作为SPE吸附剂,建立了尿液中6种羟基多环芳烃的前处理方法(见图6)。GO的引入增强柱床与羟基多环芳烃的*π-π*共轭作用,将萃取效率由空白柱的70.7%~86.3%提升至94.2%~101%。该方法简单、高效,为尿液中羟基多环芳烃的分析提供了新方法,为复杂样品基质中羟基多环芳烃的前处理提供了新思路。Wang等^[71]^在注射器中构筑亚氨基多孔金属有机笼掺杂聚EDMA整体柱,结合高效液相色谱-串联质谱法,分析藜麦样品中的蜕皮类固醇。掺杂整体柱比表面积由47.0 m^2^/g提升至153.8 m^2^/g,蜕皮类固醇与吸附剂的作用主要为疏水、*π-π*、范德华力和氢键作用。

**图6:**
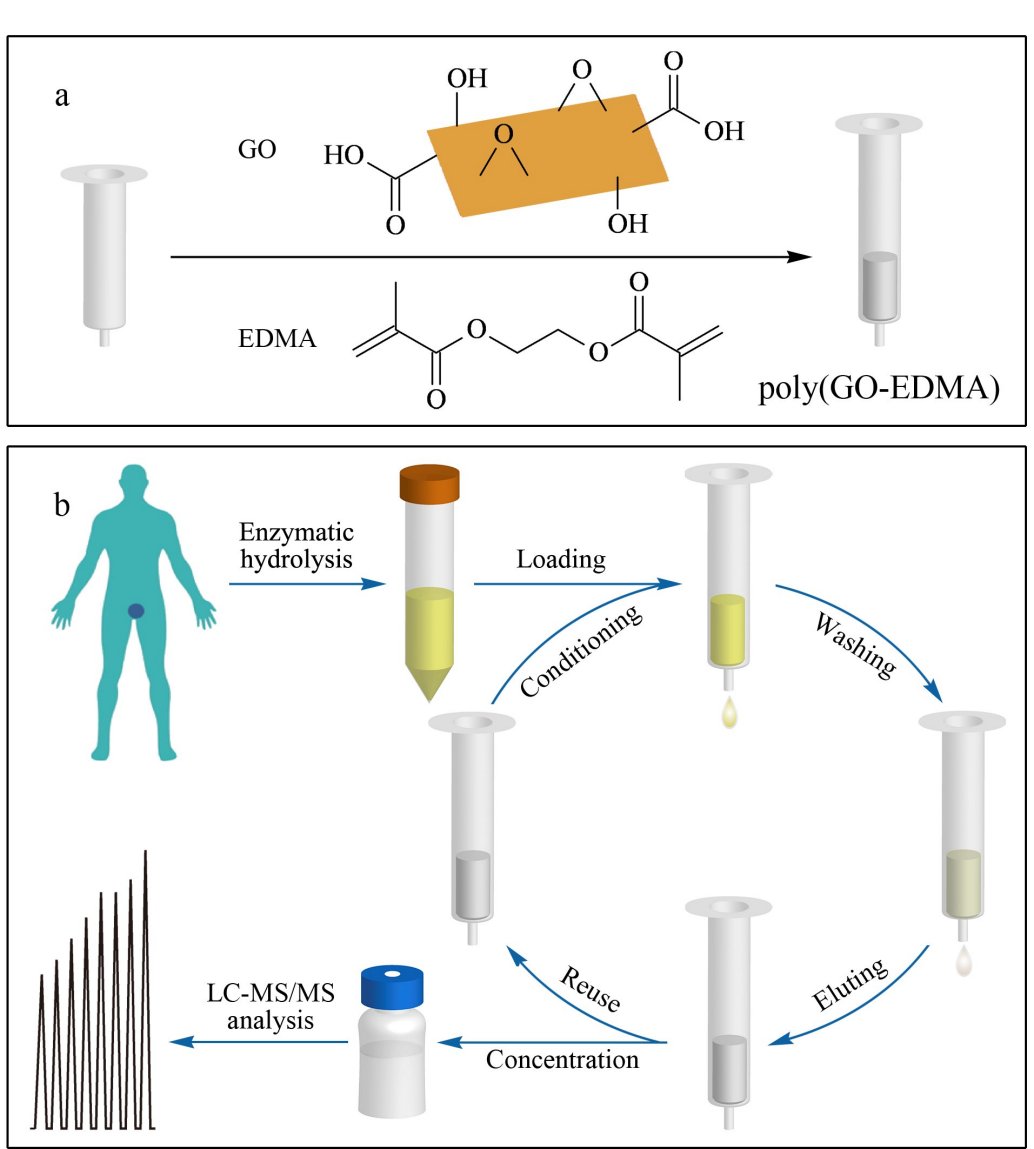
(a)聚(GO-EDMA)整体柱的构筑和(b)聚(GO-EDMA) 整体柱SPE前处理尿液中羟基多环芳烃流程图^[70]^

### 3.2 固相微萃取

SPME是以某种纤维或者材料作为载体,在其表面涂覆不同性质的吸附材料薄膜,通过直接或顶空微萃取的方式,实现对样品的吸附、进样和脱附,具有操作简单、萃取速度快、有机溶剂消耗少且环境友好等优点,可实现对痕量目标物集取样、萃取、浓缩和进样于一体的绿色前处理方法^[72,73]^。SPME涂层决定了萃取的选择性、容量和速度,从而影响整个分析方法的灵敏度和使用范围。基于纳米材料掺杂的整体柱具有高吸附容量和高通透性能等优点,近年来已成为广泛应用的SPME涂层材料。

Fresco-Cala等^[74]^以GMA为单体、EDMA为交联剂,光引发制备的碳纳米角掺杂的聚合物整体柱作为SPME吸附剂预处理尿液中4种非甾体抗炎药。该柱光引发10 min即可完成制备,简单快速。相比空白柱的比表面积(<10 m^2^/g),掺杂碳纳米角的整体柱比表面积显著增加至700 m^2^/g,同时增强了整体柱的机械强度和化学稳定性,并通过*π-π*和氢键作用吸附目标物,检出限为0.1~10 μg/L。Luo等^[59]^通过热引发成功制备聚(NH_2_-MIL-53-St-DVB-MAA)整体柱作为SPME涂层用于尿液中雌激素的前处理,掺杂MIL-53的整体柱与雌激素之间存在疏水、*π-π*和氢键作用,可有效富集目标物达到180~304倍,检出限为2.0~40 ng/L,方法无须进一步的净化程序,简便且灵敏。Lirio等^[75]^采用微波辅助法将BMA、EDMA以及不同比例的铝基金属有机骨架MIL-53聚合,制备聚(MIL-53-BMA-co-EDMA)整体柱,作为SPME吸附剂前处理河水和牛奶中青霉素。MIL-53与青霉素通过*π-π*和氢键作用,使得方法检出限低(0.060~0.26 μg/L),且线性范围宽(30~3000 μg/L)。

### 3.3 搅拌棒吸附萃取

SBSE是在SPME基础上建立的一种绿色、环保的样品前处理技术,主要采用浸没方式将涂层与样品直接接触从而吸附目标分析物,具有操作简便、溶剂用量少、富集倍数高和环境友好等优点^[76,77]^。但搅拌棒在搅拌过程中与容器的接触会造成磨损,降低使用寿命。纳米材料掺杂的整体柱可以提高柱床的机械强度和稳定性,从而有效萃取目标物,近年来作为SBSE涂层备受青睐。

You等^[78]^将ZIF-8、单体和致孔剂均匀混合,通过一锅法制备聚(ZIF-8-甲基丙烯酸甲酯-co-EDMA)整体柱用于SBSE前处理水果中5种植物激素。ZIF-8的引入增大了整体柱的比表面积,与植物激素之间的静电、疏水、氢键和*π-π*共轭作用,使得整体柱的萃取效率(48%~57%)明显高于空白柱(4%~32%)和商品化SBSE吸附剂(2%~37%)。Yang等^[79]^制备UiO-66掺杂的聚合物整体柱作为SBSE涂层,用于土壤和湖水中5种磺酰脲类除草剂的分析。UiO-66通过氢键和*π-π*共轭与磺酰脲类除草剂作用,使得聚合物整体柱对目标物的萃取效率提升3.7~4.5倍。

### 3.4 在线固相萃取

在线固相萃取是将固相萃取净化柱作为第一维色谱柱完成上样、淋洗、洗脱及条件化等步骤后,将富集净化后的目标物通过切换阀转移至第二维色谱柱的分析柱上,完成目标物的分离与分析。在线固相萃取操作简单,避免离线操作过程中样品损失和遭受污染,减小操作误差,分析速度快^[80,81]^。在线固相萃取净化柱是整个在线净化系统的核心。聚合物整体柱制备简单,可以在毛细管柱、不锈钢柱和移液枪枪尖等多种模具中构筑,便于与仪器联用。纳米掺杂的整体柱同时兼具聚合物和纳米材料的双重作用,将其与在线固相萃取技术结合可实现固相萃取净化和分离分析的自动化。

杨成雄等^[82]^将MIL-101掺杂的聚合物整体柱用于在线SPE环境水中4种酚类化合物。MIL-101加入后,在*π-π*共轭和氢键相互作用下,提高了整体柱的富集能力,富集因子为71~127。Cui等^[83]^以1-辛烯(1-octene, C8)为单体,三甘醇二甲基丙烯酸酯(TEGDA)为交联剂,通过原位自由基共聚法制备GO掺杂的聚合物整体柱,用于在线固相萃取食用油中*β*-谷甾醇。GO的掺杂使整体柱对目标物的吸附容量(82.5 mg/g)明显高于空白柱(34.6 mg/g)。同时该课题组^[84]^采用纳米金刚石掺杂的聚合物整体柱,在线固相萃取食物油中的*β*-谷甾醇。加入纳米金刚石后,聚合物整体柱的比表面积由4.96 m^2^/g提升至20.97 m^2^/g,耐受的最高温度(300 ℃)高于空白柱(245 ℃)。

综上,纳米材料掺杂聚合物整体柱的制备和应用见表1。

**表1:** 纳米材料掺杂的聚合物整体柱的制备和应用

Comonomer system		Nanomaterial		Initiation	Pretreatment mode		Sample			Analyte	Ref.
GMA/EDMA		MWCNT		UV/30 min	SPME		urine			antidepressants	18
St/EDMA		MWCNT		30 ℃/3 h	on-line SPE		medicinal plants			ursolic acid	19
MAA/EDMA		MWCNT		AIBN/60 ℃/24 h	SPME		cosmetic and personal care products			parabens	20
BzMA/EDMA		CNT		AIBN/70 ℃/20 h	SPE		water			polycyclic aromatic hydrocar- bons	69
GMA/EDMA		carbon nanohorns		UV	SPE		urine			non-steroidal anti-inflammatory drugs	74
BMA/EDMA		G		AIBN/60 ℃/48 h	SPME		cosmetic			glucocorticoids	23
Comonomer system	Nanomaterial		Initiation			Pretreatment mode		Sample	Analyte		Ref.
VP/EDMA	G		AIBN/70 ℃/12 h			SPME		water and rice	phenoxyacetic acid herbicides		24
GMA/EDMA	GO		AIBN/60 ℃			SPME		urine	sarcosine		27
MAA/EDMA	GO		AIBN/65 ℃/24 h			SPME		environmental water	carbamate insecticides		28
EDMA	GO		AIBN/60 ℃/30 h			on-line SPE		milk and chicken muscle	sulfonamides		29
EDMA	GO		AIBN/65 ℃/25 h			SPE		urine	hydroxyl polycyclic aromatic hydrocarbons		70
C8/TEGDA	GO		30 ℃/2.5 h			on-line SPE		edible oil	*β*-sitosterol		83
BMA/EDMA	AC		AIBN/microwave/900 W/5 min			SPME		drinking water	phthalate esters		32
BMA/EDMA	AC		AIBN/microwave/900 W/5 min			SPME		fruit wine and cranberry juice	phenolic acid		33
GMA-EDMA	AuNPs		UV/10 min			SPE		saliva and urine	glutathione		35
GMA-EDMA	AuNPs		AIBN/60 ℃/24 h			SPME		blood plasma	glutathione		62
UF	AgNPs		70 ℃/10 min			SPME		french fries	monounsaturated fatty acid methyl esters		36
MAA-GMA/ EDMA	In_2_O_3_		AIBN/60 ℃/20 h			SPME		food	synthetic colorants		38
MAA/EDMA	ZnO		AIBN/60 ℃/24 h			SPME		environmental water	fluoroquinolone antibiotics		39
NIPAAm/ GMA/EDMA	γ-Al_2_O_3_		65 ℃/16 h			PMME		red wine	sudan		41
MAA/EDMA	γ-Al_3_O_4_		AIBN/UV/3 h			SPME		chlorzoxazone tablets	2-amino-4-chlorophenol		58
BMA/EDMA	MIL-53 (Al)		AIBN/60 ℃/12 h			SPME		water and urine	non-steroidal anti-inflammatory drugs		45
NMA/EDMA	MOF-199 (Al)		30 ℃/3.5 h			on-line SPE		Chinese herbal medicine	ursolic acid		46
BMA/EDMA	MIL-101 (Cr)		AIBN/microwave/900 W/5 min			SPME		river water	penicillin		47
St/DVB/MAA	MIL-53 (Al)		AIBN/70 ℃/24 h			on-line SPME		urine	estrogens		59
GMA/EDMA	MIL-101 (Cr)		UV			SPME		urine	nonsteroidal anti-inflammatory drugs		60
BMA-EDMA	MIL-53 (Al)		AIBN/microwave/ 900 W/5 min			SPME		river water and milk	penicillins		75
MMA/EDMA	ZIF-8		AIBN/60 ℃/24 h			SBSE		fruit	phytohormones		78
VP/EDMA	UiO-66 (Zr)		AIBN/55-60 ℃/ 3 days			SBSE		water and soil	sulfonylurea herbicides		79
GMA/EDMA	MIL-101		AIBN/70 ℃/24 h			on-line SPE		environmental water	phenols		82
GMA/EDMA	COF		AIBN/60 ℃/20 h			SPME		urine and serum	benzophenones		50
St-divinyl/ GMA	COF		120 ℃/72 h			SPE		environmental water	non-steroidal anti-inflammatory drugs		51
UF	HAP		70 ℃/2 h			SPE		grass carp	adenosine triphosphate and its phosphorylated metabolites		57
EDMA	porous organic cage		AIBN/60 ℃/12 h			SPE		chenopodium quinoa willd	ecdysteroids		71
TEGDA	nanodiamond		30 ℃/2.5 h			on-line SPE		edible oil	*β*-sitosterol		84

UV: ultraviolet; AIBN: alpha-azo-isobutyronitrile; AuNPs: gold nanoparticles; NMA: *N*-methylolacrylamide; UF: urea-formaldehyde; AgNPs: silver nanoparticles; NIPAAm: *N*-isopropylacrylamide; PMME: polymer monolith microextraction; MMA: methyl methacrylate.

## 4 结论与展望

目前,对于纳米材料掺杂聚合物整体柱,通过简单掺杂、共聚合和原位修饰等合成方法,可以显著提高聚合物整体柱的选择性和稳定性等性能,同时改善其微观结构和吸附性能。另一方面,纳米材料掺杂聚合物整体柱萃取模式多种多样,涵盖SPE、SPME、SBSE和在线SPE等多种样品前处理技术,丰富了复杂样品的萃取模式,促进样品前处理领域的发展。此外,纳米材料掺杂聚合物整体柱已广泛应用于食品安全和环境卫生领域低浓度风险化合物的前处理,同时,凭借纳米材料掺杂聚合物整体柱的表面特异性和生物兼容性,使得该材料对生物样品和天然药物中的目标化合物具有高选择性的富集能力。然而,该类材料仍然会受到纳米材料性质和合成条件的限制,例如对纳米材料的粒径、密度和合成的溶剂等条件有一定要求;另外在材料的均匀性和稳定性方面仍有待进一步改善。充分发挥并挖掘纳米材料掺杂聚合物整体柱的优势,在今后的研究中继续发展新的纳米材料的种类,发展多样的合成方法,探索制备和吸附分离机理并进一步拓展探索该类材料在生物医药、食品分析和环境健康等领域的应用,对于卫生检验、材料科学等多领域协同发展以及实际分析问题的有效解决具有重要意义。
